# A multiparameter model predicting in-hospital mortality in malignant cerebral infarction

**DOI:** 10.1097/MD.0000000000007443

**Published:** 2017-07-14

**Authors:** Chien-Fu Chen, Ruey-Tay Lin, Hsiu-Fen Lin, A-Ching Chao

**Affiliations:** a Department of Neurology, Kaohsiung Medical University Hospital; b Department of Neurology, College of Medicine, Kaohsiung Medical University, Kaohsiung, Taiwan.

**Keywords:** brain edema, CHADS_2_ score, large hemisphere infarction, malignant cerebral infarction, middle cerebral artery

## Abstract

The early identification of patients with large hemisphere infarctions (LHIs) at risk of fatal brain edema may result in better outcomes. A quantitative model using parameters obtained at admission may be a predictor of in-hospital mortality from LHI.

This prospective study enrolled all patients with LHI involving >50% of the middle cerebral artery (MCA) admitted to our neurological intensive care unit within 48 hours of symptom onset. Early clinical and radiographic parameters and the baseline CHADS_2_ score (congestive heart failure, hypertension, age ≥ 75 years, diabetes mellitus, stroke [double weight]) were analyzed regarding their ability to predict patient outcomes.

Seventy-seven patients with LHIs were identified, 33 (42.9%) with complete MCA infarction (CMCA), and 44 (57.1%) with incomplete MCA infarction (IMCA). The predictors of CMCA score included: >1/3 early hypodensity in computed tomography findings, hyperdense MCA sign, brain edema, initial National Institutes of Health Stroke Scale (NIHSS) score ≥17, and stroke in progression during the 1st 5 days of admission. The cutoff CMCA score was 2, with a sensitivity of 81.8% and specificity of 70.5%. Mortality score 1, used for predicting in-hospital mortality from LHI, included CMCA and CHADS_2_ scores ≥4 (sensitivity 100.0%, specificity 57.4%), and mortality score 2 included CMCA and CHADS_2_ scores ≥4, and NIHSS score ≥26, during the 1st 5 days (sensitivity 100.0%, specificity 91.7%).

Patients qualifying for a mortality score of 2 were at high-risk of in-hospital mortality from LHI. These findings may aid in identifying patients who may benefit from invasive therapeutic strategies, and in better describing the characteristics of those at risk of mortality.

## 1 Introduction

Large hemispheric infarcts (LHIs) account for only 3% to 15% of all ischemic infarcts, but are associated with high mortality and morbidity, including a reported in-hospital mortality rate of 17% to 80%.^[1–10]^ Malignant cerebral infarction (MCI) resulting from space-occupying brain edema after an ischemic stroke in the middle cerebral artery (MCA) territory has been reported to result in a mortality rate of approximately 80% under conservative treatment.^[2]^ These large cerebral infarctions often result in severe shifting of midline structures, with subsequent uncal or even transtentorial herniation, and thus have been associated with a poor prognosis in more than 80% of cases.^[4,11–13]^

Various factors have been reported to be independent predictors of MCI, including vomiting, hypertension, congestive heart failure, and infarct volume.^[4,14–19]^ Atrial fibrillation (AF) is an important cause of massive, fatal cerebral infarctions in the elderly.^[20]^ The CHADS_2_ score (congestive heart failure, hypertension, age ≥ 75 years, diabetes mellitus, stroke [double weight]) is a simple classification system that is used to estimate the risk of ischemic stroke in patients with AF.^[21]^ However, it has also been reported to successfully predict all-cause mortality after stroke, and to provide a valuable insight into other outcome variables which can be applied to patients with different risk factor profiles, such as those with a known cardiovascular disease other than AF.^[22]^

The association between CHADS_2_ scores and in-hospital mortality from MCI has yet to be investigated. However, the early identification of high-risk patients with MCI who may be suitable for (and respond to) aggressive intervention is critical. The main goal of this study was to investigate the predictors of clinical, radiographic, and baseline CHADS_2_ score for a malignant course in a prospectively evaluated population with large MCA territory infarcts. The rationale for this was to provide clinicians caring for patients with acute stroke with early prognostic parameters that may aid in deciding the most appropriate treatment for these patients.

## 2 Patients and methods

### 2.1 Study design

We prospectively investigated patients admitted to the neurological intensive care unit of Kaohsiung Medical University Hospital because of acute LHI within 48 hours after stroke onset. From February 2010 to July 2011, a total of 77 patients with LHI were identified and included in this study. The protocol was approved by the Ethics Committee of the Medical Faculty of Kaohsiung Municipal Hsiaokang Hospital, and informed consent was obtained from each participant or a legal representative. LHI was defined as an infarct involving at least 1/2 (deep, superior, and posterior) of the MCA territories^[3]^ in either a 2nd computed tomography (CT) or magnetic resonance imaging (MRI) scan. Determination of vascular distribution was based on the anterior circulation vascular territory templates provided by Tatu et al.^[23]^ Mass effect and infarct size were determined by grading hemispheric swelling.^[24]^ Complete MCA infarction (CMCA) was defined as the involvement of all 3 (deep, superior, and posterior) MCA territories, and incomplete MCA infarction (IMCA) was defined as an infarct involving more than 50% of the MCA territories.

Demographic data including age, sex, hypertension, known diabetes mellitus, cigarette smoking, hypercholesterolemia, and a history of cardiac disease including congestive heart failure, AF, coronary artery disease, and a history of previous stroke or transient ischemic attack were recorded. Laboratory results of serum glucose, complete blood count, fibrinogen, troponin I, HbA1C, prothrombin time, and partial activated thrombin time were included for analysis. Each patient was assessed for stroke severity using the National Institute of Health Stroke Scale (NIHSS).^[25]^ Clinical outcomes were assessed according to stroke in progression and in-hospital mortality. All patients received an initial brain CT scan and/or MRI study, and emergency follow-up CT studies were also performed if significant neurological deterioration occurred. A 2nd brain CT scan and/or MRI study was performed within 5 days after admission. Stroke in progression was defined as an increase in NIHSS score by 2 or more points (or stroke-related death) between admission and day 5.^[26]^

Original CT scans were reviewed by a stroke neurologist blinded to follow-up imaging and clinical details. Early signs of massive infarction were interpreted according to the methods of Kasner et al and Davalos et al.^[4,27]^ The early CT signs evaluated included: hyperdense MCA sign (HMCAS) defined as spontaneous high contrast in the MCA brighter than adjacent brain tissue and other intracranial arteries (particularly the contralateral MCA) not attributable to calcification^[28]^; >1/3 early hypodensity defined as the extent of focal hypodensity in the MCA territories (>33%); and brain edema defined as sulcal effacement, mass effect, midline shift, or ventricular compression. Sulcal effacement included: lentiform obscuration defined as a loss of the precise delineation of the lentiform nucleus because of a decrease in density compared with the contralateral nucleus^[29]^; and Sylvian fissure obscuration defined as effacement of the Sylvian fissure compared with the contralateral side.^[24]^

### 2.2 Outcome measures

CMCA score was defined as the predictive score for CMCA, and included the following parameters: >1/3 early hypodensity in initial CT findings; HMCAS; brain edema; NIHSS score at admission ≥17; and stroke in progression during the 1st 5 days after admission. Mortality score 1 was defined as the 1st predictive score for in-hospital mortality of the stroke patients, and included CMCA and a CHADS_2_ score ≥4, with the clinical and radiographic predictors each being assigned 1 point. Mortality score 2 was defined the 2nd predictive score for in-hospital mortality and included CMCA and CHADS_2_ scores ≥4, and NIHSS score ≥26 on day 5 after admission. Each of the clinical and radiographic predictors was assigned 1 point.

### 2.3 Statistical analysis

Values were expressed as means ± standard deviation. Comparisons of numerical and categorical variables between the patients with CMCA and IMCA were conducted using 2-sample *t* tests and chi-square tests, respectively. Receiver-operating characteristic (ROC) curves and areas under the curves (AUCs) were plotted and calculated to compare the predictive accuracy for morbidity and mortality of large MCA infarctions.^[30]^ All data analyses were performed using JMP software (SAS Institutes Inc., Cary, NC).

## 3 Results

### 3.1 Characteristics of the patients in the CMCA and IMCA groups

Demographic and clinical features are summarized in Table 1. There were 77 patients, including 41 men and 36 women, with a mean (±SD) age of 70.7 ± 12.6 years (range 40–94 years). Forty-four (57.1%) of the patients had IMCAs, and 33 (42.9%) had CMCAs. The most common risk factor for stroke was hypertension (68.8%). Seventeen patients (22.1%) had stroke in progression within 5 days after admission. There were no significant differences in age, gender, body mass index, baseline blood pressure, associated cardiovascular risk factors, blood glucose, white blood cell count, fibrinogen, or troponin I between the patients with CMCA and IMCA. The patients with CMCA had a significantly higher ratio of stroke in progression within 5 days after admission than the patients with IMCA (33.3% vs 13.6%; *P* = .04). The patients with CMCA had a higher mean NIHSS score both at admission (20.3 vs 15.6; *P* *<* .001) and on day 5 (20.8 vs 14.6; *P* *<* .001) than the patients with IMCA. The patients with CMCA had a significantly higher ratio of in-hospital mortality than the patients with IMCA (24.2% vs 2.3%; *P* = .03). A similar number of patients were treated with thrombolysis in both groups. Baseline CT characteristics showed significantly greater early involvement of >1/3 of the MCA territories (48.8% vs 15.9%, *P* = .002), HMCAS (54.55% vs 25%, *P* = .008), and early brain edema (54.55% vs 29.55%, *P* = .03) in the patients with CMCA than in those with IMCA. However, there was no significant difference in hemorrhagic transformation between the patients with CMCA and IMCA. Three patients with CMCA underwent decompressive craniectomy, 2 of them died after decompressive craniectomy. In contrast, no patients with IMCA underwent decompressive craniectomy and 1 patient with IMCA died on hospital discharge.

**Table 1:**
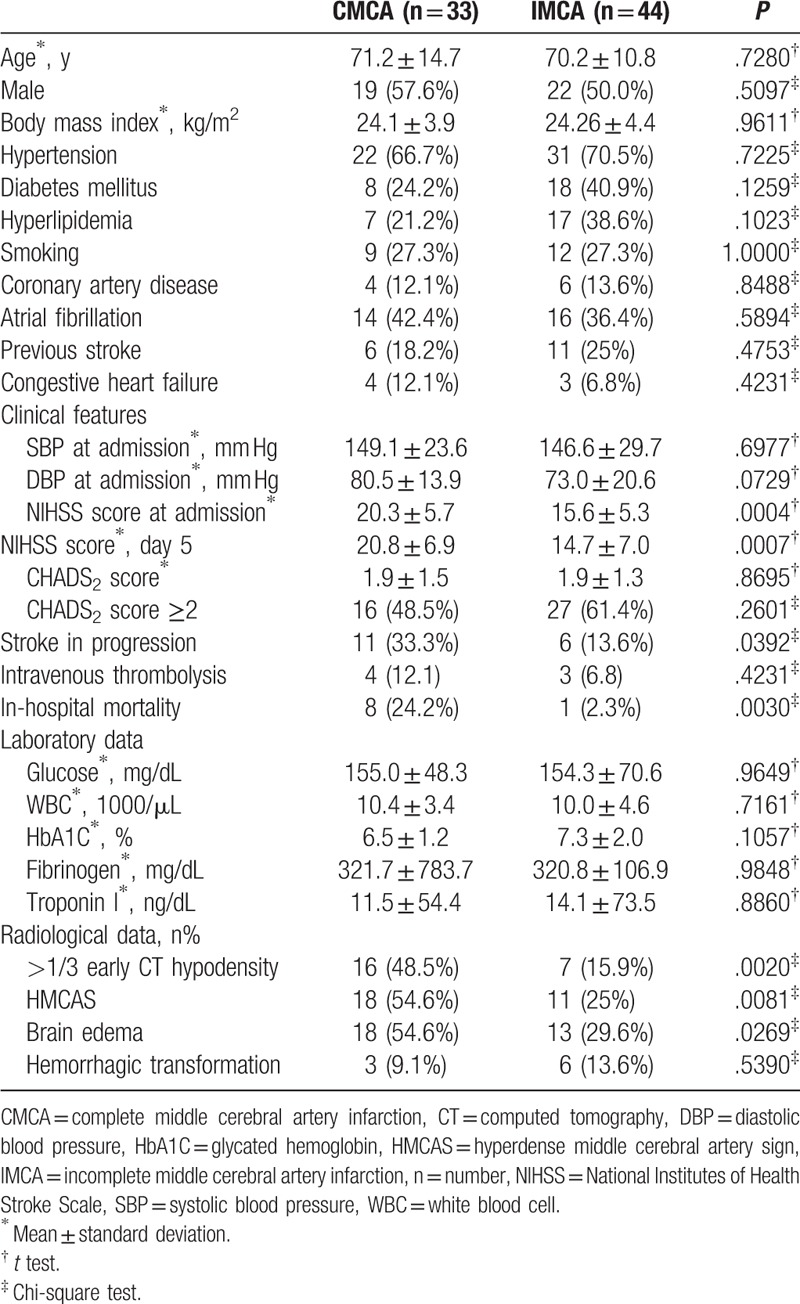
Demographic and clinical features of the patients with stroke.

Table 2 shows the basic data, clinical courses, and early CT findings of 9 (11.7%) patients who died in-hospital, 8 of whom had CMCA. The NIHSS score ranged from 17 to 29 at admission, and 15 to 35 at day 5 after admission. Five of the patients had AF, and the CHADS_2_ score was 3 or more in 6 patients. Decompressive hemicraniectomy was performed in 2 of the 9 patients. Only 3 of the 9 patients had early CT findings of >1/3 MCA hypodense lesions, HMCAS, and brain edema at admission.

**Table 2:**
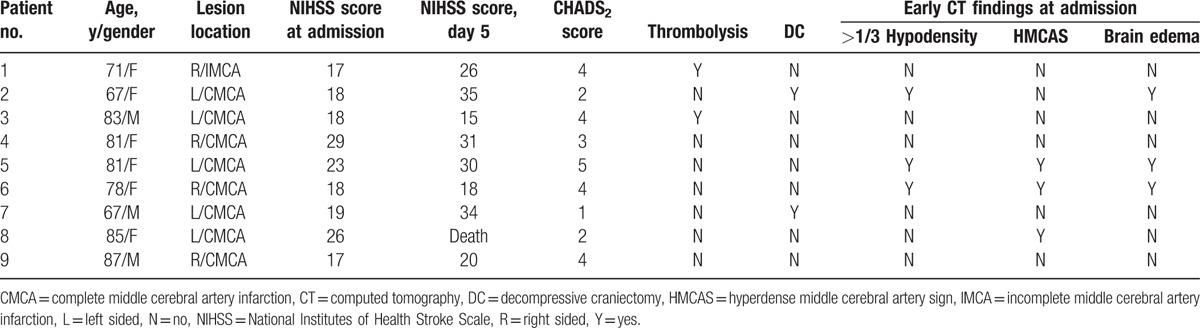
Summary of data from all patients who died in-hospital.

### 3.2 Predictors of in-hospital mortality

**Figure 1:**
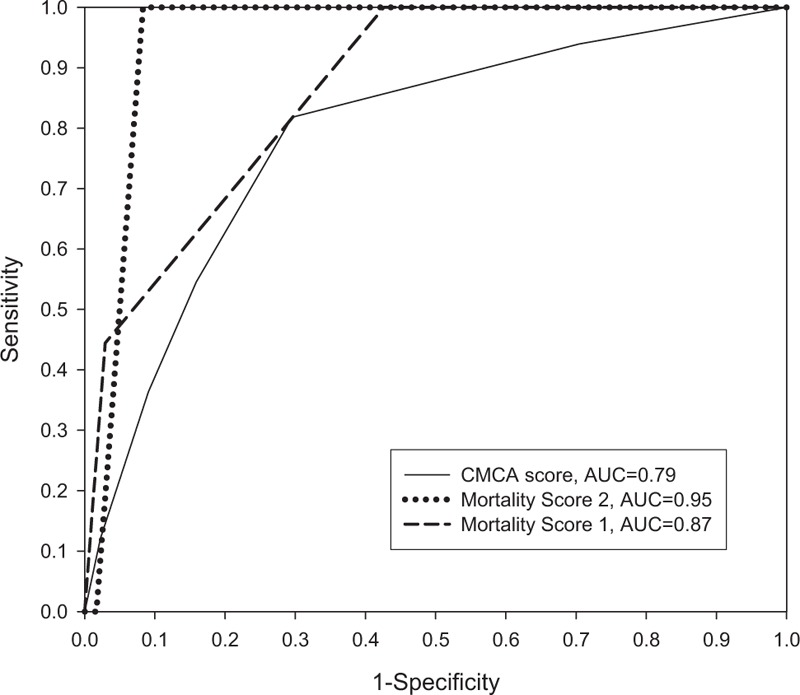
The predictive accuracy of measurements in CMCA and mortality score for overall stroke patients, using ROC curves and areas under the ROC curves. AUC = area under the curve, CMCA = complete middle cerebral artery infarction, ROC = receiver-operating characteristic.

To further evaluate the predictors of morbidity and mortality for large MCA infarctions, ROC curves were plotted and the AUCs are shown in Table 3 and Fig. 1. In general, the AUC is a reasonable summary of the overall diagnostic accuracy of a continuous variable.

**Table 3:**
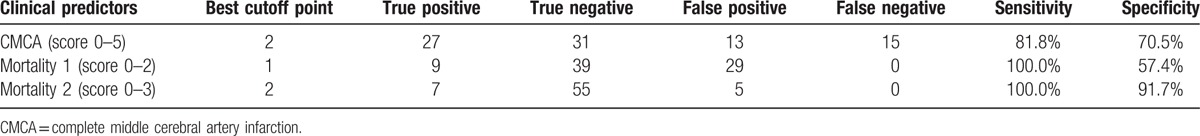
The cutoff points of clinical predictors on each receiver-operating characteristic curve.

**Figure 2:**
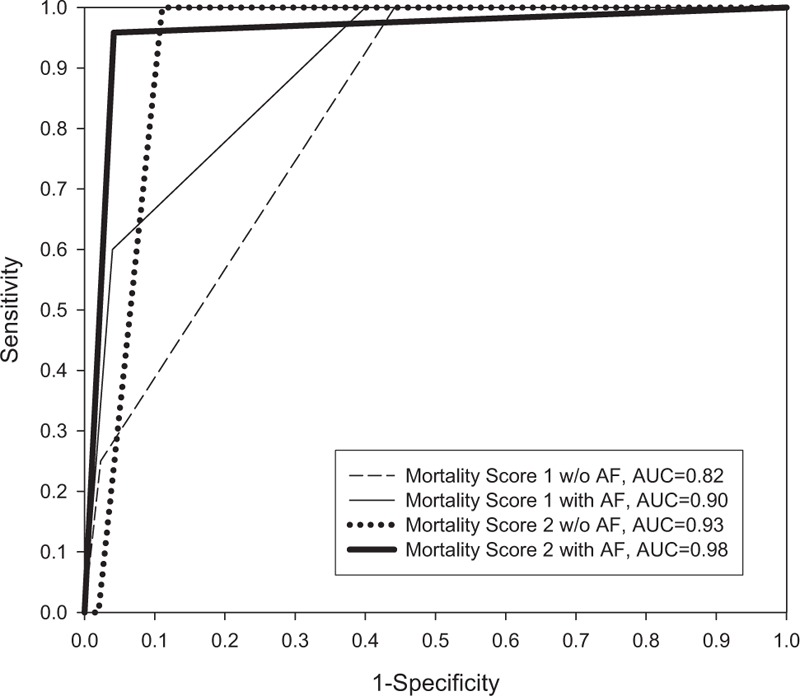
Comparison of predictive accuracy of measurements in mortality score between patients with and without atrial fibrillation.

From the ROC curves (Fig. 1) of the clinical parameters, mortality score 2 had the highest AUC to predict in-hospital mortality (AUC = 0.95). Mortality score 1 also had a high AUC to predict in-hospital mortality from a large MCA stroke (AUC = 0.87). The CMCA risk score was also a good early predictor of a large MCA infarction (AUC = 0.79). In ROC curve analysis, the cutoff point that yielded the best combination of sensitivity and specificity was 2 (sensitivity 100.0%; specificity 91.7% for mortality score 2, Table 3). For mortality score 1, the cutoff point was 1, with a sensitivity of 100.0% and specificity of 57.4%. For the occurrence of CMCA, the cutoff point was 2 (sensitivity 81.8%; specificity 70.5%). The comparison of the predictive accuracy of measurements in the mortality score between patients with and without AF is shown in Fig. 2. The AUC of mortality score 1 was 0.82, compared to 0.90 between patients without and with AF. The AUC of mortality score 2 was 0.93, compared to 0.98 between patients with and without AF. These findings imply that LHI has an impact on all-cause in-hospital mortality, and that the proposed risk classification system can be applied to patients with different risk factor profiles, such as those with a previously identified cardiovascular disease but without known AF.

## 4 Discussion

In the present study, we used clinical and radiological data and the baseline CHADS_2_ scores to derive a model based on patient characteristics available at the time of hospital admission that could predict in-hospital mortality from MCI. This model generated a CMCA score that incorporated initial CT findings and stroke severity to predict an individual's risk of dying in hospital after admission for stroke. In addition, we proposed a 2nd predictive model similar to the 1st model, but incorporating CHADS_2_ and NIHSS scores, to predict in-hospital mortality from LHI. To the best of our knowledge, this is the first study to investigate the relationship between CHADS_2_ score and MCI. The CHADS_2_ score assesses the cardiovascular risk of cardiac embolic stroke in patients with AF. A CHADS_2_ score ≥4 was associated with a high risk of fatal brain edema or life-threatening complications in this study.

The term “malignant” was first used by Hacke et al^[2]^ to characterize the complete infarction of MCA territories accompanied by a space-occupying mass effect that develops during the first 5 days after presentation, and that is associated with a mortality rate of about 80%. In general, a neuroradiological definition of a malignant MCA infarction assumes that at least two thirds of the MCA-perfused territories are affected. In addition, patients with >50% MCA territory involvement have a high risk of malignant change.^[2,18]^ Brain CT is widely used to diagnose and monitor patients with a malignant MCA infarction, although those findings regarding the MCA infarction gathered on the 1st or 2nd day are not definite enough to make a diagnosis of CMCA. Therefore, we sought to identify clinical and radiographic factors that could predict MCI among patients with large MCA territory infarcts using early CT findings with at least 50% MCA territory involvement. We assessed the associated clinical and radiographic parameters of the CMCA score, including >1/3 early hypodensity; HMCAS; brain edema; NIHSS score at admission ≥17; and stroke in progression during the 1st 5 days after admission. To identify CMCA, the cutoff score was 2, with a sensitivity of 82% and specificity of 70%. With this model, stroke physicians may be able to make an early prediction of the infarct size or stroke severity within 24 to 48 hours of admission, if 2 of these 5 radiographic and clinical parameters are present in patients with an MCA infarction at admission. Because of the higher potential risk in these patients of fatal brain edema and life-threatening complications, we believe such an early predictive ability would provide for superior treatment and improved outcomes.

Malignant edema in humans is difficult to predict on the 1st day after stroke.^[31]^ In patients with a malignant MCA infarction, decompressive surgery performed within 48 hours of stroke onset reduced mortality and improved functional outcomes in a pooled analysis of 3 randomized controlled trials.^[32–34]^ However, repeated brain CT for the 1st 3 days after stroke onset may be necessary to determine the true extent of infarction, as well as any associated brain swelling and midline shift. These limitations prevent a prompt decision on whether or not to perform decompressive craniectomy. In this study, we tried to find a simple method to allow for making an early diagnosis of CMCA using available clinical and radiological parameters. The CMCA score model may help with that regard. In addition, mortality scores 1 and 2 could then be used to evaluate the risk of the development of malignant evolution after a large hemispheric stroke. The 1st predictive score for in-hospital mortality was mortality score 1, calculated on the 1st day after admission. Its parameters, predictive for a large MCA stroke, included the CMCA score and a CHADS_2_ score ≥4. In short, patients with CMCA or patients with IMCA with a CHADS_2_ score ≥4 had a high risk of mortality. The 2nd predictive score, for in-hospital mortality within 5 days after admission, was mortality score 2. Its predictive parameters included CMCA, a CHADS_2_ score ≥4, and an NIHSS score ≥26 during the 1st 5 days after admission. Patients with 2 of these 3 parameters during the 1st 5 days after admission are at high risk of fatal brain edema or associated life-threatening cardiovascular complications. Hence, stroke physicians may be able to use the 3-risk scoring system to make an early prediction for patients with LHIs.

This study was limited by its relatively small sample size, and further validation of these models in studies with a larger sample size of patients should be conducted in the future. In addition, future research is needed to examine how this risk score can be used, and its potential utility in (and effect on) clinical practice. It will also be necessary to determine the characteristics associated with differences in observed versus expected mortality rates, and to determine whether accurate risk scores can be developed for other stroke types.

## 5 Conclusion

In conclusion, we identified several readily available clinical and radiographic features that may be useful in predicting which patients admitted with a large MCA stroke are at high risk of mortality. We also found an association between CHADS_2_ scores and in-hospital mortality from a large MCA stroke, in that a CMCA, a CHADS_2_ score ≥4, or an NIHSS score ≥26 within the 1st 5 days after admission all predicted fatal brain edema or life-threatening complications. The 3 predictive risk scores – CMCA score, mortality score 1 and mortality score 2 – may provide clinicians caring for acute stroke patients with early prognostic parameters that may help with decisions regarding treatment.

## Acknowledgments

The authors thank assistance from the Statistical Analysis Laboratory, Department of Clinical Research, Kaohsiung Medical University Hospital, Taiwan.
